# Supplementary material for: Basic Needs in Horses?—A Literature Review
Source: Animals (Basel). 2021 Jun 16;11(6):1798. doi: 10.3390/ani11061798 (PMC8235049; doi:10.3390/ani11061798)
Supplement: Supplementary file 1 [file animals-11-01798-s001.zip › File S1 Statistical Data.pdf]

Basic needs in horses? - A literature review

Konstanze Krueger[1,2]*, Laureen Esch[1,3], Kate Farmer[4], Isabell Marr[1,5]

[1] Nuertingen-Geislingen University, Faculty Agriculture, Economics and Management, Department Equine Economics, Neckarsteige 6-10, 72622 Nürtingen, Germany

[2] University of Regensburg, Zoology / Evolutionary Biology, Universitätsstraße 31, 93053 Regensburg, Germany

[3] Ludwig Maximilian University Munich, Veterinarian Medicine, Department of animal welfare, ethology, animal hygiene and animal husbandry, Veterinaerstr. 13/R, 80539 Munich, Germany

[4] Centre for Social Learning & Cognitive Evolution, School of Psychology, University of St Andrews, St Andrews, Scotland KY16 9JPh, Germany

[5] University of Hohenheim, Behavioural Physiology of Farm Animals, Garbenstr. 17, 70599 Hohenheim, Germany

* **Corresponding author:** Nuertingen-Geislingen University, Faculty Agriculture, Economics and Management, Department Equine Economics, Neckarsteige 6-10, 72622 Nürtingen, Germany, Tel: +497022201331, Email: Konstanze.krueger@hfwu.de

File S1: Statistical Data. Complete Generalized Linear Models (GLMs).

Generalized Linear Model(GLM). General effects

```
Call:
glm(formula = nr. of ms. evaluating response ~ response versus no response +
    response type, family = poisson(identity), data = Dataset)

Deviance Residuals:
    Min       1Q   Median       3Q      Max
-2.3928  -1.3176  -0.7996   0.0804   3.6523

Coefficients:
                            Estimate Std. Error z value  Pr(>|z|)
(Intercept)                   2.1830     0.5269   4.143 0.0000343 ***
response versus no response   1.5171     0.3723   4.075 0.0000461 ***
response type                -0.4187     0.1630  -2.569    0.0102 *
---
Signif. codes:  0 '***' 0.001 '**' 0.01 '*' 0.05 '.' 0.1 ' ' 1

(Dispersion parameter for poisson family taken to be 1)

    Null deviance: 134.16  on 50  degrees of freedom
Residual deviance: 112.29  on 48  degrees of freedom
  (21 observations deleted due to missingness)
AIC: 210.46

Number of Fisher Scoring iterations: 5
```

General Linear Model (GLM). Response nested in response type

```
Call:
glm(formula = nr. of ms. evaluating response ~ response versus no response %in%
    response type, family = poisson(identity), data = Dataset)

Deviance Residuals:
    Min       1Q   Median       3Q      Max
-2.3452  -1.4936  -0.8726   0.0249   3.7415

Coefficients:
                                           Estimate Std. Error z value     Pr(>|z|)
(Intercept)                                  1.1154     0.2071   5.385 0.0000000724 ***
response versus no response:type_abnormal   1.2179     0.6571   1.854      0.06381 .
response versus no response:type_active     1.6346     0.6218   2.629      0.00857 **
response versus no response:type_passive    1.0275     0.5908   1.739      0.08200 .
response versus no response:type_stress     2.3846     0.9581   2.489      0.01281 *
---
Signif. codes:  0 '***' 0.001 '**' 0.01 '*' 0.05 '.' 0.1 ' ' 1

(Dispersion parameter for poisson family taken to be 1)

    Null deviance: 134.16  on 50  degrees of freedom
Residual deviance: 116.61  on 46  degrees of freedom
  (21 observations deleted due to missingness)
AIC: 218.77
```
